# Evaluation of DrugWipe^®^ 6S with the WipeAlyser^®^ reader for drug screening of drivers

**DOI:** 10.1093/jat/bkaf028

**Published:** 2025-04-11

**Authors:** Ragnhild Elén Gjulem Jamt, Hallvard Gjerde, Grethe Brennhovd Clausen, Lihn Bache-Andreassen, Elisabeth Leere Øiestad

**Affiliations:** Department of Forensic Sciences, Oslo University Hospital, PO Box 4950, Nydalen, Oslo 0424, Norway; Department of Forensic Sciences, Oslo University Hospital, PO Box 4950, Nydalen, Oslo 0424, Norway; Technical and Material Department, Norwegian National Road Policing Service, PO Box 100, Stavern 3291, Norway; Department of Forensic Sciences, Oslo University Hospital, PO Box 4950, Nydalen, Oslo 0424, Norway; Department of Forensic Sciences, Oslo University Hospital, PO Box 4950, Nydalen, Oslo 0424, Norway; Department of Pharmacy, University of Oslo, PO Box 1068 Blindern, Oslo 0316, Norway

## Abstract

On-site drug screening of oral fluid samples has gained attention because of its convenience and rapid results. The aim of this investigation was to compare the results of preliminary screening for drugs in oral fluid samples collected from suspected drug-impaired drivers using DrugWipe 6S and WipeAlyser reader with the results obtained from blood samples. Additionally, we compared the DrugWipe test results with findings of drug traces detected within the used DrugWipe devices. Police officers selected a sample of 355 suspected drug-impaired drivers in 2023. They used DrugWipe 6S for preliminary drug screening of drivers. After the field drug testing of oral fluid, the apprehended drivers were brought to a physician for the collection of blood samples. The collected samples (DrugWipe devices and blood samples) were submitted to the Norwegian National Forensic Toxicology Laboratory for analysis. The proportion of positive DrugWipe results that were unconfirmed when analysing blood samples was 82% for opiates, 75% for cocaine, and ∼19%–20% for amphetamines, cannabis, and benzodiazepines. The proportion of negative DrugWipe results that were found positive in blood samples was for cannabis and benzodiazepines ∼13%–14%, and for other drugs <3%. Detected drug traces in the used DrugWipe devices corresponded well with DrugWipe readouts for cannabis, amphetamines, and cocaine. The lack of correspondence between DrugWipe test results for cocaine and findings in blood may be due to the fact that the concentration of cocaine in saliva is often much higher than in blood, and the DrugWipe test is very sensitive. In addition, degradation and elimination of cocaine before the blood sample is taken may contribute to cocaine concentrations below the cut-off concentration in blood. For opiates and benzodiazepines, traces of drugs were found in relatively few DrugWipe devices. Many unconfirmed positives for opiates were most likely due to cross-reaction with substances in ‘snus’ (snuff tobacco).

## Introduction

Drug-impaired driving presents a significant hazard to road safety [1–3]. To address this issue, numerous jurisdictions have enacted legislation aimed at preventing and penalizing drug-impaired driving. Several countries have impairment laws requiring documentation of signs of clinical impairment in addition to a positive drug test result to convict a driver of drug-impaired driving, and some jurisdictions have adopted zero-tolerance legislation on the use of illicit drugs before driving [1, 4]. Other jurisdictions have established legal limits for drug concentrations in blood [5].

In many countries, law enforcement officers may stop drivers for control of alcohol and drug use when they observe hazardous driving behaviours, such as speeding, weaving, and running red lights. They may also look for alcohol or drug impairment among crash-involved drivers and in cases where bystanders report possible driving under the influence. In many jurisdictions, including Norway, they may also perform random controls of drivers. When alcohol-impaired driving is suspected, a hand-held alcohol breath screening test may be used to confirm or dismiss this suspicion. In instances of suspected drug impairment, an on-site oral fluid drug screening device may be used for preliminary drug screening [6, 7]. In some jurisdictions, field sobriety tests are used to strengthen the suspicion of impairment [8].

On-site (point-of-collection) drug screening of oral fluid samples has gained attention because of its convenience and rapid results. Additionally, the collection of oral fluid is less intrusive than urine or blood collection. Immediate results assist law enforcement in assessing impairment and, in some jurisdictions, in obtaining search warrants for further testing. However, for legal proceedings, laboratory-based confirmation using chromatographic-mass spectroscopic analytical techniques remains essential. Confirmation and determination of drug concentration are performed by analysing blood samples in most jurisdictions. Nevertheless, a few countries with zero-tolerance legislation, such as Australia, Belgium, France, and Spain, may employ oral fluid samples for confirmation analysis [4]. Notably, a positive test result for listed illicit drugs in oral fluid is sufficient for conviction in these countries.

The detection of drugs in oral fluid confirms recent intake, but detection windows after the last use vary significantly for different drugs [9, 10]. Some drugs may only be detectable within hours, whereas others persist for days. In addition to drug type, the duration of the detection window also depends on factors such as the dosage, frequency of use, route of administration, and the cut-off concentration used by the analytical methods or drug screening device.

The proportion of unconfirmed positives (UPs; positive DrugWipe test and negative blood confirmation) and unconfirmed negatives (UNs; negative DrugWipe test and positive blood confirmation) depends on the specificity of the analytical method, the cut-off concentrations used when analysing oral fluid and blood samples, the drug concentration ratio between oral fluid and blood, and the time gap between the DrugWipe test and the collection of blood samples. False positives may occur when using antibody-based screening devices because of cross-reactions with drugs and metabolites with similar chemical structures and sometimes with substances unrelated to the drugs being tested for, such as chemicals in coffee and food, chewing tobacco, and ‘snus’ (finely ground tobacco placed in the mouth) [11, 12]. In addition, random errors may produce false positive drug screening results.

It is important to note that the drug concentration in oral fluid cannot be used to assess the degree of impairment or intoxication or the drug concentration in blood even when analysed using confirmatory methods. This discrepancy arises because the drug concentration ratio between oral fluid and blood varies significantly among individuals [13, 14].

In Norway, legal drug concentration limits in blood were implemented for drivers in 2012, with sanctions corresponding to those given for driving with blood alcohol concentrations >0.2, 0.5, and 1.2 g/kg [15]. The police were also authorized to use roadside preliminary drug screening tests. Since 2015, the Norwegian police has performed roadside screening of drugs in oral fluid from drivers suspected of driving under the influence of drugs (DUID). During the first years, Dräger DrugTest 5000 (DDT5000) instruments were used (manufactured by Drägerwerk AG & Co., Lübeck, Germany), while DrugWipe 6S instruments combined with WipeAlyser readers (both manufactured by Securetec Detektions-Systeme AG, Neubiberg/Munich, Germany) have been used since 2020.

A previous study has been conducted on the effectiveness of DDT5000 in a police context to detect drug-impaired drivers in Norway [16]. The study revealed large proportions of positive DDT5000 results that were unconfirmed when analysing blood samples for cocaine (81%) and opiates (66%). Interestingly, traces of those substances were often detected in oral fluid samples collected simultaneously with the blood samples in cases of positive DDT5000 and negative blood test results, indicating presence in oral fluid. For benzodiazepines and amphetamines, the proportion of UPs ranged from 23% to 36%. Cannabis had the lowest proportion of UPs. The proportion of UNs was the highest for benzodiazepines (19%) followed by cannabis (14%). Despite these limitations, law enforcement authorities reported that DDT5000 was a valuable tool for identifying potential DUID offenders.

**Figure 1.**
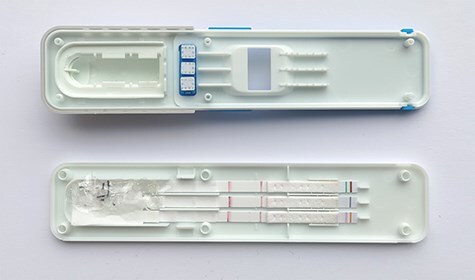
A used DrugWipe device.

The DrugWipe device uses a lateral flow immunoassay to screen for the presence of drugs in oral fluid. Oral fluid is collected by swiping the collector pads on the tongue until the colour of the device’s three sampling pads turns yellow. About 30 µl oral fluid is collected: ∼10 µl for each collection pad, but the volume may vary (personal communication between Elisabeth Leere Øiestad and Securetec) (Fig. 1). The sample collector then transfers the sample to test strips containing antibodies specific to certain drug classes. If the sample contains drugs, they will bind to the antibodies, producing a red line after a few minutes. The test result can be inspected visually, or by using the WipeAlyser reading device.

The objective of this investigation was to compare the results (positive or negative) of preliminary drug screening for drugs in oral fluid samples collected from suspected DUID offenders using DrugWipe 6S with the results obtained from blood samples. Additionally, we compared the DrugWipe test results with findings of drug traces detected within the used DrugWipe devices.

## Materials and methods

### Collection of samples

Norwegian National Road Policing Service officers selected a sample of suspected drug-impaired drivers from mid-February to the end of June 2023. The selection was conducted during the planned routine control for alcohol- or drug-impaired driving in road traffic but did not constitute a random or systematic selection. It may therefore be regarded as a convenience sample. They employed the DrugWipe 6S testing device with the WipeAlyser reader for preliminary drug screening of drivers as a part of their routine control. A reporting sheet was filled in with DrugWipe test results. The driver was brought to a physician for the collection of blood samples as required by the Norwegian Road Traffic Act. Whole blood was collected in 5 ml Vacutainer® tubes containing 20 mg sodium fluoride and 143 I.U. heparin (BD Vacutainer Systems, Belliver Industrial Estate, Plymouth, UK). The used DrugWipe device, the reporting sheet, and the blood sample were labelled with the same barcode and submitted by mail or transported by car directly to the Norwegian National Forensic Toxicology Laboratory in Oslo (Department of Forensic Sciences, Oslo University Hospital). The DrugWipe devices were stored at −20°C and blood samples at 2°C–8°C until analysed.

### Laboratory analysis

#### Oral fluid samples

The DrugWipe screening device (Fig. 1) was opened, and both the collection pads and cotton pads on the test strips were transferred to a 15-ml glass vial. Subsequently, 3 ml of a mixture of acetonitrile and water containing 0.25% NH_3_ (75:25, v/v) was added to the glass vial as recommended by the manufacturer (personal communication from W. Sooth at Securetec to the study supervisor). In addition, 100 µl of Triton-X 500 and 50 µl of the internal standard (for the list of included substances, see Supplementary Table A) were added. After 5 min of mixing and 5 min of centrifugation (3500 rpm), 2 ml supernatant was transferred to a new glass vial and 25 µl of methanol containing 0.01% HNO_3_ was added. The sample was then evaporated to dryness and reconstituted in 100 µl of a mixture of isopropanol and acetonitrile (50:50, v/v). Following centrifugation for 5 min, 80 µl of the reconstituted sample was transferred to an injection vial. The samples were analysed using ultra high-performance liquid chromatography–tandem mass spectrometry (UHPLC–MS-MS). A method originally developed for blood samples was adapted with a sample preparation suited for DrugWipe extracts [17].

The recovery of drugs from DrugWipe devices was studied by preparing two sets consisting of five samples of oral fluid each (set A and set B) (for flowchart of the recovery experiment, see Supplementary Fig. A). In set A, oral fluid samples of 600 µl each were spiked with 10 µl of a standard solution containing the drugs listed in Table 1. Then, 10 µl of the oral fluid-standard solution mixture was added on each collection pad of five DrugWipe devices. In set B, 10 µl of oral fluid was applied to each collection pad of five DrugWipe devices. Sets A and B were stored in the refrigerator until the next day, before both sets A and B were prepared and extracted according to the procedure described in the paragraph above. After the evaporation step, 50 µl of the internal standard was added to each vial of sets A and B. Then, 50 µl of isopropanol and acetonitrile (50:50, v/v) was added to each of the five vials of set A. For set B, a mixture consisting of 10 µl of a standard solution containing the drugs listed in Table 1 and 1 ml of isopropanol and acetonitrile (50:50, v/v) was prepared. Then 50 µl of this mixture was added to the five vials of set B. All vials were centrifuged for 5 min, and finally, 80 µl was transferred to an injection vial. The samples were analysed using UHPLC–MS-MS. Recovery was calculated as follows:


$$\% {\mathrm{Recovery}} = \left( {\frac{{{P_{\mathrm{A}}}}}{{{P_{\mathrm{B}}}}}} \right) \times 100$$

where *P*_A_ depicts the peak area of a compound in a sample spiked before extraction (set A) and *P*_B_ depicts the peak area of the same compound in a sample spiked after extraction (set B).

**Table 1.** Cut-off concentrations for the DrugWipe screening device and the methods using UHPLC–MS-MS

	Cut-off concentrations of analytical methods		
Compound	DrugWipe 6S screening^a^ (ng/ml)	Extract from DrugWipe^b^ (ng/ml)	Blood^c^ (ng/ml)
CNS stimulants			
Amphetamine	50	50	41
Methamphetamine	50	50	45
3,4-Methylenedioxymethamphetamine	NS	50	97
Cocaine	10	10	24
Benzoylecgonine	25	25	23^d^
Benzodiazepines and similar substances			
Alprazolam	5	5	3
Bromazepam	10	NA	32
Diazepam	5	11	57
Etizolam	NS	NA	14
Phenazepam	NS	NA	1.8
Flunitrazepam	10	NA	1.6
Clobazam	100	NA	180
Clonazepam	20	51	1.3
Lorazepam	50	NA	9.6
Nitrazepam	10	38	17
Nordiazepam	5	22	108
Oxazepam	100	192	172
Cannabis			
Δ^9-^Tetrahydrocannabinol (THC)	10	10	1.3
Opiates/opioids			
Codeine	5	8	100^d^
Morphine	10	29	9

CNS = central nervous system; NS = not stated; NA = not analysed.

a According to the manufacturer.

b Concentration in the collected oral fluid.

c Legal limits.

d Legal limit not assigned.

#### Blood samples

Preparation of blood samples was conducted with a Tecan Freedom EVO liquid robot system (Tecan Group Ltd, Männedorf, Switzerland). An aliquot of 100 µl of each sample of whole blood was transferred to a test tube, and 50 µl internal standard solution and 100 µl ethanol:water mixture (30:70, v/v) were added consecutively with mixing. Samples were then transferred to a 96-well Nunc 2-ml well plate (Thermo Fischer Scientific, Waltham, MA, USA). To precipitate proteins, 400 µl ice-cold mixture of acetonitrile and methanol (85:15, v/v) was added to each well, followed by centrifugation of the 96-well plate. Next, an aliquot of 420 µl of each supernatant was transferred to a Captiva EMR-lipid filterplate (Agilent Technologies, Santa Clara, CA, USA) after adding 200 µl acetonitrile:water with 1% formic acid (80:20, v/v) to each well. Filtration was performed with vacuum.

Filtered sample extracts were analysed using a Xevo TQ-XS UHPLC–MS-MS instrument (Waters Corporation, Milford, MA, USA). Separation was performed using a Waters Aquity HSST3 column (2.1 × 100 mm, 1, 8 µm particles) with a 5-mm precolumn with the same material, employing gradient elution. Mobile phase A consisted of 10 mM ammonium formate buffer pH 3.1, while mobile phase B was methanol. Confirmation was performed according to published methods [18–21]. For the quantification of opiates and cocaine, a method originally developed for urine [22] was adapted with a sample preparation suited for blood.

The cut-off concentrations for the DrugWipe screening device, the UHPLC–MS-MS methods for oral fluid and blood are presented in Table 1. For blood samples, the legal *per se* limits for DUID were used as cut-off concentrations, if assigned. The oral fluid cut-off was based on drug concentration in spiked oral fluid calibrators giving a signal to noise ratio of 10.

### Data analysis

The ID numbers on the DrugWipe screening devices and questionnaires were matched with the results of the respective blood sample analyzes. The ID numbers were then replaced by a project specific ID number and an anonymous database was generated. The data processing was performed using SPSS 29 (IBM Corporation, Armonk, NY, USA). DrugWipe/WipeAlyser readouts were compared with the analytical results for blood and the following parameters were established: confirmed positive (CP) is a case with a positive DrugWipe result and a positive blood drug finding; confirmed negative (CN) is a case with a negative DrugWipe result and a negative blood drug finding (not detected or concentration below cut-off); a UP is a case with a positive DrugWipe result and a negative blood drug finding (not detected or concentration below the cut-off); a UN is a case with a negative DrugWipe result and a positive blood drug finding. Furthermore, we calculated sensitivity, specificity, accuracy, prevalence, positive predictive value (PPV), and negative predictive value (NPV) for each substance and drug class of the test.

## Results

We received blood samples and used DrugWipe devices with corresponding test results (readouts) from 355 drivers. In total, 343 drivers tested positive by DrugWipe; 43% of those (147 drivers) had positive readouts for two or more substance groups. Blood drug concentrations above the cut-offs were found in 306 cases; 39% of those (118 drivers) tested positive for two or more substances. The median recorded time gap between DrugWipe testing and the collection of blood samples was 35 min; the time gap was >60 min in 15% of the cases. DrugWipe test results, findings of drug traces inside the used DrugWipe devices, and drug findings above the cut-off concentrations in blood samples (the legal limits) are presented in Table 2.

**Table 2.** Test results (readouts) for saliva with DrugWipe/WipeAlyser and blood (*n* = 355)

Compound	Positive DrugWipe test (*n*)	Drug detections in DrugWipe extracts (*n*)	Drug detections in blood (*n*)
CNS stimulants			
Amphetamines	124	136	104
Amphetamine		132	101
Methamphetamine		7	2
3,4-Methylenedioxymethamphetamine			4
Cocaine	126	124	32
Benzoylecgonine		83	73
Benzodiazepines and similar drugs			
Benzodiazepines	48	39	78
Alprazolam		20	45
Bromazepam		NA	0
Clobazam		NA	0
Clonazepam		1	23
Diazepam		19	36
Etizolam		NA	1
Fenazepam		NA	0
Flunitrazepam		NA	0
Lorazepam		NA	0
Nitrazepam		0	1
Nordiazepam		7	29
Oxazepam		0	4
Cannabinoids			
Δ^9-^tetrahydrocannabinol (THC)	181	233	169
Opiates/opioids	62	21	11
Codeine		13	2
Morphine		12	9

NA = not analysed.

### Comparison of DrugWipe test results with findings from blood samples

Table 3 presents the classification into CP, UP, CN, and UN, along with the calculations of prevalence. The sensitivity, specificity, accuracy, PPV, and NPV for each substance group tested are presented in Supplementary Table B.

**Table 3.** Test results (readouts) for DrugWipe/WipeAlyser compared with the analytical results for blood samples

Drug	DrugWipe positive, *n*	Blood sample CP, % (*n*)	Blood sample CN, % (*n*)	DrugWipe negative, (*n*)	Blood sample CN, % (*n*)	Blood sample CP, % (*n*)
Cannabis	181	80 (145)	20 (36)	174	86 (150)	14 (24)
Amphetamines	124	80 (99)	20 (25)	231	98 (226)	2 (5)
Cocaine	126	25 (31)	75 (95)	229	100 (228)	0 (1)
Opiates	62	18 (11)	82 (51)	293	100 (293)	0 (0)
Benzodiazepines	48	81 (39)	19 (9)	307	87 (268)	13 (39)

The proportion of UP results was 82% for opiates, 75% for cocaine, and ∼19%–20% for amphetamines, cannabis, and benzodiazepines. The proportion of UNs was ∼13%–14% for cannabis and benzodiazepines, and <3% for other drugs.

The DrugWipe test for cocaine may cross-react with the inactive metabolite benzoylecgonine. Among the 126 cases with a positive DrugWipe readout for cocaine, benzoylecgonine was detected in 68 of the blood samples (54%), and cocaine (together with benzoylecgonine) in 31 of the blood samples (25%) (results not shown).

A total of 62 drivers tested positive for opiates by DrugWipe. Nine of these drivers (15%) had concentrations of morphine above the cut-off concentration in their blood, and two drivers exhibited high codeine concentrations in their blood.

In 12 cases, blood samples were collected despite negative DrugWipe tests. This likely occurred because the police officers suspected drug impairment even when the DrugWipe test was negative. Out of these cases, seven tested positive for drugs in blood: in four cases only THC, in two cases THC combined with other drugs, and in one case amphetamine and a benzodiazepine. In all these cases, traces of the drugs found in the blood samples were also detected in extracts from the used DrugWipe devices.

### Recovery of drugs from used DrugWipe devices

The recovery of drugs from DrugWipe devices is presented in Supplementary Table C. Overall, the recovery of drugs was low from the used DrugWipe devices, particularly for benzodiazepines, which exhibited a recovery varying from 0% for oxazepam to 8% for alprazolam. The highest recovery of all substances was found for THC, with a recovery rate of 47%. The recovery of the CNS stimulants varied between 15% and 26%, and the recovery of opiates varied between 22% and 26%.

### Comparison of DrugWipe test results with findings of drug traces in the used DrugWipe devices

Results of the analysis of extracts of DrugWipe collectors and test strip pads are presented in Table 4. The drug findings corresponded well with the DrugWipe readouts for cannabis (98%) and amphetamines (88%). Traces of cocaine were found in 105 of 126 extracts with positive DrugWipe readouts for cocaine (83%). In addition, three extracts (2%) contained traces of benzoylecgonine but not cocaine.

**Table 4.** Test results (readouts) for DrugWipe/WipeAlyser compared with analytical results for extracts from used DrugWipe devices

Drug	DrugWipe positive, (*n*)	Extract CP, % (*n*)	Extract CN, % (*n*)	DrugWipe negative, (*n*)	Extract CN, % (*n*)	Extract CP, % (*n*)
Cannabis	181	98 (177)	2 (4)	174	70 (122)	30 (52)
Amphetamines	124	88 (109)	12 (15)	231	90 (209)	10 (22)
Cocaine	126	83 (105)	17 (21)	229	94 (215)	6 (14)
Opiates	62	24 (15)	76 (47)	293	98 (287)	2 (6)
Benzodiazepines	48	48 (23)	52 (25)	307	95 (292)	5 (15)

In 95 cases, the DrugWipe readout indicated cocaine, whereas the blood samples tested negative for cocaine. Extracts of those DrugWipe devices tested positive for cocaine in 75 cases (79%). In addition, two extracts (2%) contained traces of benzoylecgonine but not cocaine. For opiates and benzodiazepines, traces of drugs were found in relatively few DrugWipe devices.

Traces of THC were found in 52 of 174 extracts from DrugWipe devices with a negative result (30%). The proportion of similarly UN cases was lower for other drugs.

## Discussion

When conducting confirmation analysis in blood samples, the proportions of UP results for DrugWipe were the highest for opiates and cocaine. However, detected drug traces in the used DrugWipe devices corresponded well with DrugWipe readouts for cannabis, amphetamines, and cocaine, indicating that those substances had been used even if the blood samples tested negative. Similar results were reported by Gentili *et al*., who also studied drug traces in used DrugWipe devices [23].

The most likely reasons for not finding cocaine above the cut-off concentration in blood when the DrugWipe device showed a positive result were (i) the concentration of cocaine in saliva is often much higher than in blood and the DrugWipe test is very sensitive; (ii) the degradation and elimination of cocaine in blood between apprehension on the road and the collection of blood samples is quick; and (iii) multiple dosing and high exposure to cocaine may lead to accumulation in deep body compartments, prolonging the presence of cocaine in oral fluid [24, 25]. In some high-exposure cases, the concentration of cocaine might be lower than the cut-off concentration in blood but above the cut-off concentration in oral fluid many hours after the last cocaine use. The main reason for a lack of correspondence between test results for cocaine in oral fluid and blood is thus not related to the DrugWipe device itself.

Also for opiates, there were many cases where the blood samples tested negative, while the DrugWipe device produced a positive test result. In addition, extracts from opiate-positive devices most often yielded negative results for opiates. The underlying reason for this high incidence of unconfirmed opioid findings remains unclear. Police experience suggests that it in many cases may be caused by cross-reaction with substances found in ‘snus’, a smokeless tobacco product commonly used in Norway. Notably, the manufacturer of DrugWipe has confirmed that the use of ‘snus’ can lead to false positive test results for opiates (personal communication from a Securetec representative to the police). Furthermore, we have also found that consuming an orange immediately before testing with the DrugWipe device may also result in a positive readout for amphetamines and opiates (unpublished observation by Grethe Brennhovd Clausen and Ragnhild Elén Gjulem Jamt).

Traces of benzodiazepines were rarely detected in extracts from DrugWipe devices. The primary reason for this limited detection was likely related to the small amount of oral fluid collected by the DrugWipe device and the very low concentrations of benzodiazepines in oral fluid as compared with those in blood [13, 14]. Additionally, the recovery of drugs when extracting residual drugs from the device was poor. Consequently, the drug concentrations in these extracts were often lower than the cut-off for the analytical method used for DrugWipe extracts. The comparison between used DrugWipe devices and blood therefore suffers from a significant limitation.

Better data may be obtained if using an optimized extraction procedure for drug traces in the used DrugWipe devices. In general, a better correspondence between DrugWipe^®^ 6S results and oral fluid would be expected to be found if the on-site results had been compared to a sample collected with a dedicated oral fluid collection device, taken at the same time. We have previously demonstrated this in a study exploring the performance of the Dräger 5000 device [16]. This was however not possible to include in the present study.

For cannabis, amphetamines, and benzodiazepines, there were few cases in which positive DrugWipe readouts were followed by negative test results for blood samples (see Supplementary Table B). Positive readouts for these substances thus indicated a high probability of detecting these substances in blood.

In cases where DrugWipe readouts were negative for amphetamines, cocaine, and opiates, >97% of these cases also tested negative in blood. Consequently, negative readouts for these substances indicated a low probability of testing positive in blood. However, for cannabis and benzodiazepines, ∼12%–13% of the cases with negative DrugWipe readouts exhibited concentrations of these drugs above the cut-off concentrations in blood.

Although we have compared results of drug findings in oral fluid to the findings in blood, this is suboptimal, as we are comparing the findings in two different biological matrices that were not collected at the same time. Consequently, the calculated sensitivity, specificity, accuracy, PPV, and NPV for each substance group have inherent limitations and should be interpreted with this in mind. We have therefore presented those data only in Supplementary Table B.

Table 5 presents an overview of previously published studies that compared DrugWipe test results with those obtained from blood or serum/plasma samples. Overall, the proportion of UP findings was the highest for cocaine and opiates, consistent with this study. Conversely, UNs were the most common for cannabis and amphetamines. Other studies did not find a similar proportion of UNs for benzodiazepines as we found; however, the numbers are small and therefore less reliable. It is essential to recognize that the proportions of true UP and UN test results vary across studies because of factors such as sample type (blood or serum/plasma), cut-off concentrations used for DrugWipe and blood, prevalence in the studied population, time between DrugWipe testing and blood sample collection, and the specific types of amphetamines, benzodiazepines, and opiates detected. Studies that compared DrugWipe test results with confirmation analysis of oral fluid generally found higher proportions of true positives and true negatives (see Supplementary Table D) than studies comparing DrugWipe results with findings in blood.

**Table 5.** DrugWipe results compared with results for blood samples

Drug class	References	Cut-off concentration in blood/serum (ng/mL)	DrugWipe positive, (*n*)	Blood sample CP, % (*n*)	Blood sample CN, % (*n*)	DrugWipe negative, (*n*)	Blood sample CN, % (*n*)	Blood sample CP, % (*n*)
Cannabis	Liut *et al*. [26]	1^a^	471	81 (382)	19 (89)	442	85 (374)	15 (68)
	Wille *et al*. [27]	1^a^	24	88 (21)	12 (3)	48	56 (27)	44 (21)
	Musshoff *et al*. [28]	1^a^	17	71 (12)	29 (5)	7	29 (2)	71 (5)
	Pehrsson *et al*. [29]	1	293	29 (84)	71 (209)	1514	93 (1402)	7 (112)
	Wille *et al*. [30]	1^a^	29	69 (20)	31 (9)	17	53 (9)	47 (8)
	Pehrsson *et al*. [31]	1	55	51 (28)	49 (27)	210	94 (197)	6 (13)
Amphetamines	Liut *et al*. [26]	25^a^	388	72 (281)	28 (107)	525	96 (506)	4 (19)
	Musshoff *et al*. [28]	25^a^	13	69 (9)	31 (4)	2	100 (2)	0
	Pehrsson *et al*. [29]	25	1609	91 (1460)	9 (149)	198	75 (149)	25 (49)
	Wille *et al*. [30]	10^a^	25	84 (21)	16 (4)	6	100 (6)	0
	Pehrsson *et al*. [31]	20	222	97 (216)	3 (6)	44	89 (39)	11 (5)
Cocaine	Liut *et al*. [26]	10/75^a^	170	35 (59)	65 (111)	743	99 (737)	1 (6)
Benzoylecgonine	Musshoff *et al*. [28]	10/75^a^	6	50 (3)	50 (3)	2	100 (2)	0
	Pehrsson *et al*. [29]	10/10	38	32 (12)	68 (26)	1769	100 (1762)	0 (7)
	Wille *et al*. [30]	5^a^	14	100 (14)	0	20	80 (16)	20 (4)
	Pehrsson *et al*. [31]	20/20	6	33 (2)	67 (4)	260	99 (258)	1 (2)
Opiates	Liut *et al*. [26]	10^a^	54	39 (21)	61 (33)	859	100 (856)	0 (3)
	Musshoff *et al*. 28]	10^a^	2	50 (1)	50 (1)	1	100 (1)	0
	Pehrsson *et al*. [29]	1–10	22	18 (4)	81 (18)	1785	98 (1749)	2 (36)
	Pehrsson *et al*. [31]	1–10	15	47 (7)	53 (8)	251	100 (250)	0 (1)
Benzodiazepines	Liut *et al*. [26]	Trace^a^	10	0	100 (10)	903	100 (901)	0 (2)
	Pehrsson *et al*. [31]	5–50	56	89 (50)	11 (6)	65	62 (40)	28 (25)

a Plasma or serum.

A study similar to the present one has been conducted using DDT5000 [16]. In our investigation of DrugWipe, we observed a larger proportion of UP test results in oral fluid compared with findings in blood samples for cannabis and opiates and a lower proportion of UP test results for cocaine and benzodiazepines. For amphetamine, the findings were similar.

Some jurisdictions have implemented legal limits for selected illicit drugs in oral fluid. That type of legislation reduces the number of cases where positive test results obtained with DrugWipe or similar screening devices are unconfirmed.

## Conclusion

The analysis of oral fluid with DrugWipe (or other instruments) does, in general, not accurately reflect the drug concentration in blood. Following drug use, differences between concentrations in oral fluid and blood may result in positive oral fluid test results, even in cases where the blood sample is considered negative. In addition, some other commonly used substances can yield false positive results in the DrugWipe test. The proportion of UP test results compared with findings in blood was the highest for opiates and cocaine. Other studies have also reported a large proportion of UP test results for cannabis. In most cases with a positive DrugWipe test result for cocaine, extracts of the device showed traces of cocaine, confirming its use, even if the blood sample tested negative. Oral fluid screening for drugs is a valuable tool for identifying recent drug use; however, it is less suitable for detecting drivers with drug concentrations in blood above the legal limits. However, it can still be a valuable tool for the police. When used in cases of suspected drug-impaired driving, law enforcement officers should be informed about the limitations and should use test results in combination with signs and symptoms of drug use and other relevant information before the collection of blood samples and suspension of the driver’s licence.

## Supplementary Material

bkaf028_Supplementary_Data

## Data Availability

We do not have permission to share these research data.
