# Supplementary material for: Evaluation of DrugWipe® 6S with the WipeAlyser® reader for drug screening of drivers
Source: J Anal Toxicol. 2025 Apr 11;49(7):442–9. doi: 10.1093/jat/bkaf028 (PMC12716461; doi:10.1093/jat/bkaf028)
Supplement: bkaf028_Supplementary_Data [file bkaf028_supplementary_data.zip › jat-24-4255-File005.docx]

# Supplementary Material

## Table A. Composition of the internal standard mixture, and concentration in the added solution

| Compound | Concentration (ng/mL) |
| --- | --- |
| *CNS stimulants* |  |
| ^13^C_6_-Amphetamine | 86 |
| ^13^C_6_-Methamphetamine | 93 |
| ^13^C_6_-MDMA | 122 |
| ^13^C_6_-Cocaine | 62 |
| ^13^C_6_-Benzoylecgonine | 177 |
|  |  |
| *Benzodiazepines and similar substances* |  |
| Alprazolam-D_5_ | 63 |
| ^13^C_6_-Diazepam | 174 |
| Flunitrazepam-D_7_ | 128 |
| ^13^C_6_-Clonazepam | 129 |
| ^13^C_6_-Nitrazepam | 57 |
| ^13^C_6_-Oxazepam | 585 |
|  |  |
| *Cannabis* |  |
| ^13^C_4_-THC | 127 |
|  |  |
| *Opiates/opioids* |  |
| ^13^C_2_-Codeine | 60 |
| Morphine-D_3_ | 61 |

**Legend to Figure A.** Flowchart of the recovery experiment. OF = oral fluid, IS = internal standard, IPA = isopropanol, ACN = acetonitrile

**Figure A**

**Table B.** Confirmed and unconfirmed (in blood) positive and negative test results in oral fluid, prevalence, sensitivity, specificity, accuracy, positive predictive values (PPV) and negative predictive values (NPV) for each substance group

|  | Cannabis | Amphet-amine | Cocaine | Opiates | Benzo-diazepines |
| --- | --- | --- | --- | --- | --- |
| Confirmed positive (n) | 145 | 99 | 31 | 11 | 39 |
| Unconfirmed positive (n) | 36 | 25 | 95 | 51 | 9 |
| Confirmed negative (n) | 150 | 226 | 228 | 293 | 268 |
| Unconfirmed negative (n) | 24 | 5 | 1 | 0 | 39 |
| Prevalence (%) | 48 | 29 | 9 | 3 | 22 |
| Sensitivity (%) | 86 | 95 | 97 | 100 | 50 |
| Specificity (%) | 81 | 90 | 71 | 85 | 97 |
| Accuracy (%) | 83 | 92 | 73 | 86 | 86 |
| PPV (%) | 80 | 80 | 25 | 18 | 81 |
| NPV (%) | 86 | 98 | 100 | 100 | 87 |

Table C. Recovery of drugs from used DrugWipe devices

| Compound | Evaluated concentration (ng/mL) | Recovery (%) |
| --- | --- | --- |
| *CNS stimulants* |  |  |
| Amphetamine | 54 | 26 |
| Methamphetamine | 60 | 18 |
| 3,4-methylenedioxymethamphetamine (MDMA) | 77 | 23 |
| Cocaine |  | 20 |
| Benzoylecgonine | 41 | 15 |
|  | 39 |  |
| *Benzodiazepines and similar substances* |  |  |
| Alprazolam |  | 8 |
| Bromazepam | 41 | NA |
| Diazepam |  | 1 |
| N-desmethyldiazepam | 76 | 4 |
| Etizolam |  | NA |
| Phenazepam |  | NA |
| Flunitrazepam |  | NA |
| Clobazam |  | NA |
| Clonazepam | 17 | 2 |
| Lorazepam |  | NA |
| Nitrazepam | 38 | 2 |
| Oxazepam | 72 | 0 |
|  | 191 |  |
| *Cannabis* |  |  |
| Δ-9-tetrahydrocannabinol (THC) | 8 | 47 |
|  |  |  |
| *Opiates/opioids* |  |  |
| Codeine |  | 26 |
| Morphine | 40 | 22 |

## NA = not analysed

**Table D.** DrugWipe results compared with confirmation analysis of oral fluid samples

| **Drug class** | **References** | **Cut-off in oral fluid (ng/mL)** | **DrugWipe positive,**  **(n)** | **True  positive,**  **% (n)** | **False  positive,**  **% (n)** | **DrugWipe negative,**  **(n)** | **True  negative,**  **% (n)** | **False negative,**  **% (n)** |
| --- | --- | --- | --- | --- | --- | --- | --- | --- |
| **Cannabis** | Tang et al. (2018) ^(1)^ | 0.5 | 7 | 100 (7) | 0 | 422 | 94 (397) | 6 (25) |
|  | Gentili et al. (2016) ^(2)^ | 0.2 | 18 | 78 (14) | 22 (4) | 65 | 46 (30) | 54 (35) |
|  | Wille et al. (2015) ^(3)^ | 5 | 26 | 100 (26) | 0 | 53 | 64 (34) | 36 (19) |
|  | Logan et al. (2014) ^(4)^ | 2 | 15 | 67 (10) | 33 (5) | 75 | 83 (62) | 17 (13) |
|  | Toennes et al. (2013)^a^ ^(5)^ | 2.4 | 157 | 99 (156) | 1 (1) | 34 | 44 (15) | 56 (19) |
|  | Strano-Rossi et al. (2012) ^(6)^ | 1 | 32 | 84 (27) | 16 (5) | 470 | 93 (439) | 7 (31) |
|  | Blencowe et al. (2011) ^(7)^ | 1 | 13 | 69 (9) | 31 (4) | 121 | 90 (109) | 10 (12) |
|  | Pehrsson et al. (2008) ^(8)^ | 2 | 23 | 52 (12) | 48 (11) | 125 | 91 (114) | 9 (11) |
|  |  |  |  |  |  |  |  |  |
| **Amphetamines** | Tang et al. (2018) ^(1)^ | 5 | 156 | 78 (122) | 22 (34) | 299 | 92 (274) | 8 (25) |
|  | Gentili et al. (2016) ^(2)^ | 1-2 | 33 | 79 (26) | 21 (7) | 50 | 86 (43) | 14 (7) |
|  | Logan et al. (2014) ^(4)^ | 10 | 6 | 50 (3) | 50 (3) | 84 | 98 (82) | 2 (2) |
|  | Strano-Rossi et al. (2012) ^(6)^ | 25 | 13 | 92 (12) | 8 (1) | 490 | 100 (490) | 0 (0) |
|  | Blencowe et al. (2011) ^(7)^ | 25 | 38 | 87 (33) | 13 (5) | 97 | 95 (92) | 5 (5) |
|  | Pehrsson et al. (2008) ^(8)^ | 25 | 129 | 99 (128) | 1 (1) | 19 | 68 (13) | 32 (6) |
|  |  |  |  |  |  |  |  |  |
| **Cocaine/Benzoylecgonine** | Tang et al. (2018) ^(1)^ | 1/1 | 3 | 100 (3) | 0 (0) | 426 | 99 (422) | 1 (4) |
|  | Gentili et al. (2016) ^(2)^ | 1.1/na | 19 | 84 (16) | 16 (3) | 64 | 88 (56) | 13 (8) |
|  | Logan et al. (2014) ^(4)^ | 10/5 | 14 | 64 (9) | 36 (5) | 76 | 99 (75) | 1 (1) |
|  | Strano-Rossi et al. (2012) ^(6)^ | 10/10 | 62 | 92 (57) | 8 (5) | 442 | 99 (436) | 1 (6) |
|  | Blencowe et al. (2011) ^(7)^ | 10/10 | 0 | 0 (0) | 0 (0) | 123 | 100 (1239 | 0 (0) |
|  | Pehrsson et al. (2008) ^(8)^ | 8/8 | 2 | 50 (1) | 50 (1) | 146 | 99 (145) | 1 (1) |
|  |  |  |  |  |  |  |  |  |
| **Opiates** | Tang et al. (2018) ^(1)^ | 5 | 238 | 92 (218) | 8 (20) | 217 | 92 (200) | 8 (17) |
|  | Gentili et al. (2016) ^(2)^ | 0.2-1.3 | 9 | 22 (2) | 78 (7) | 74 | 97 (72) | 3 (2) |
|  | Logan et al. (2014) ^(4)^ | 8 | 4 | 75 (3) | 25 (1) | 86 | 98 (84) | 2 (2) |
|  | Strano-Rossi et al. (2012) ^(6)^ | 20 | 5 | 80 (4) | 20 (1) | 502 | 99 (499) | 1 (3) |
|  | Blencowe et al. (2011) ^(7)^ | 20 | 0 | 0 (0) | 0 (0) | 135 | 99 (134) | 1 (1) |
|  | Pehrsson et al. (2008) ^(8)^ | 2-20 | 10 | 40 (4) | 60 (6) | 138 | 100 (138) | 0 (0) |

^a^Sample from the tongue

**References**

1. Tang MHY, Ching CK, Poon S, et al. Evaluation of three rapid oral fluid test devices on the screening of multiple drugs of abuse including ketamine. Forensic Sci Int 2018;286:113-20. <https://doi.org/10.1016/j.forsciint.2018.03.004>

2. Gentili S, Solimini R, Tittarelli R, et al. A Study on the Reliability of an On-Site Oral Fluid Drug Test in a Recreational Context. J Anal Methods Chem 2016;2016:1234581. <https://doi.org/10.1155/2016/1234581>

3. Wille SMR, Di Fazio V, Toennes SW, et al. Evaluation of Δ9-tetrahydrocannabinol detection using DrugWipe5S® screening and oral fluid quantification after Quantisal™ collection for roadside drug detection via a controlled study with chronic cannabis users. Drug Test Anal 2015;7:178-86. <https://doi.org/10.1002/dta.1660>

4. Logan BK, Mohr AL, Talpins SK. Detection and prevalence of drug use in arrested drivers using the Dräger Drug Test 5000 and Affiniton DrugWipe oral fluid drug screening devices. J Anal Toxicol 2014;38:444-50. <https://doi.org/10.1093/jat/bku050>

5. Toennes SW, Schneider K, Wunder C, et al. Influence of ethanol on the pharmacokinetic properties of Δ9-tetrahydrocannabinol in oral fluid. J Anal Toxicol 2013;37:152-8. <https://doi.org/10.1093/jat/bkt002>

6. Strano-Rossi S, Castrignanò E, Anzillotti L, et al. Evaluation of four oral fluid devices (DDS®, Drugtest 5000®, Drugwipe 5+® and RapidSTAT®) for on-site monitoring drugged driving in comparison with UHPLC-MS/MS analysis. Forensic Sci Int 2012;221:70-6. <https://doi.org/10.1016/j.forsciint.2012.04.003>

7. Blencowe T, Pehrsson A, Lillsunde P, et al. An analytical evaluation of eight on-site oral fluid drug screening devices using laboratory confirmation results from oral fluid. Forensic Sci Int 2011;208:173-9. <https://doi.org/10.1016/j.forsciint.2010.11.026>

8. Pehrsson A, Gunnar T, Engblom C, et al. Roadside oral fluid testing: comparison of the results of drugwipe 5 and drugwipe benzodiazepines on-site tests with laboratory confirmation results of oral fluid and whole blood. Forensic Sci Int 2008;175:140-8. <https://doi.org/10.1016/j.forsciint.2007.05.022>
