# Supplementary figures and images for: Evaluation of DrugWipe® 6S with the WipeAlyser® reader for drug screening of drivers
Source: J Anal Toxicol. 2025 Apr 11;49(7):442–9. doi: 10.1093/jat/bkaf028 (PMC12716461; doi:10.1093/jat/bkaf028)

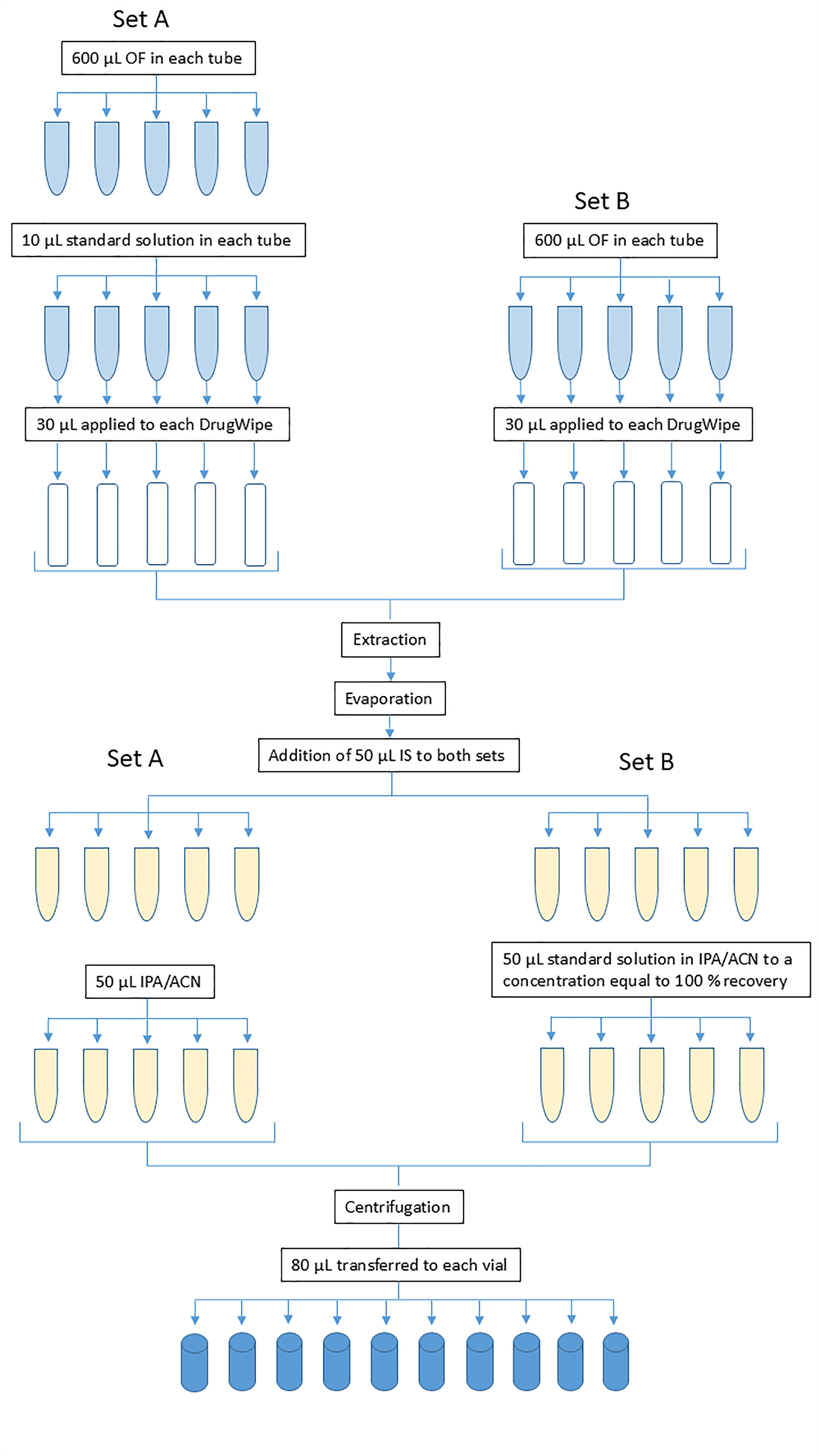

Supplement: bkaf028_Supplementary_Data [file bkaf028_supplementary_data.zip › jat-24-4255-File004.tiff]
